# Internal Secondary Relaxation as a Dielectric Probe of Molecular Surroundings

**DOI:** 10.1021/acs.jpclett.4c00128

**Published:** 2024-02-28

**Authors:** Marzena Rams-Baron, Alfred Błażytko, Maria Książek, Joachim Kusz, Marian Paluch

**Affiliations:** August Chelkowski Institute of Physics, University of Silesia in Katowice, 75 Pulku Piechoty 1, 41-500 Chorzow, Poland

## Abstract

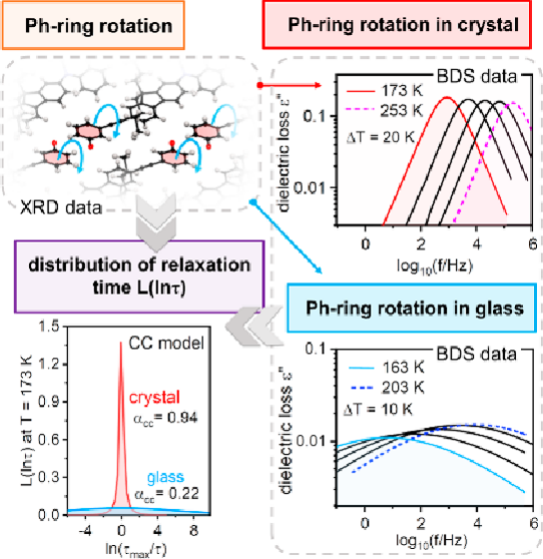

We investigated the secondary relaxation behavior in rotor molecules in a glassy and crystalline state by using the dielectric method. Without changing the molecular source of secondary relaxation, only by modifying the environment around the rotating unit we observed notable variations in spectral parameters. Our results show that internal rotation, like a probe, can sample the immediate surroundings with high sensitivity to molecular-level changes that impact the rotation parameters. Our research offers a new perspective on the dielectric behavior of internal secondary relaxations and challenges the paradigm of their irrelevant nature.

Below the glass transition temperature, *T*_g_, when the viscosity is greater than 10^12^ Pa·s and the cooperative molecular rearrangements are slower than the time scale of the experiments, secondary relaxations are the only source of information about the molecular dynamics.

Secondary relaxation processes can have basically two molecular origins and, thus, can be classified as intermolecular and intramolecular.^1−3^ Interestingly, much less attention is paid to the processes with intramolecular origin, describing them as simple, trivial, and without much relevance to the challenges faced by the glassy community.^4,5^ Intramolecular secondary relaxations governed by internal barriers and originating from side group motions are interesting from the point of view of exploring the dynamics of glass-formers with different molecular architectures, but due to the lack of connection with the fundamental glassy physics, they are definitely less deeply explored. More effort goes into intermolecular secondary relaxation research, frequently named Johari–Goldstein (JG), because only such relaxation is considered to have a strong correlation with structural relaxation and liquid-to-glass transition.^6−8,5^

In this Letter, we would like to reverse the paradigm of the lesser cognitive importance of intramolecular secondary processes. We will demonstrate that there is a special group of such relaxations in systems called molecular rotors, where internal rotation can be very sensitive to the molecular surroundings. Such behavior brings an analogy to a probe that maps certain features of the environment using internal rotations. As a consequence, the parameters of internal rotations, such as activation barriers and their distribution, differ remarkably depending on the rotor’s surroundings. To get insight into relaxation dynamics underlying internal rotations, we will use broadband dielectric spectroscopy (BDS).

The first step toward confirming the unusual behavior of internal dynamics with exceptional sensitivity to the environment was finding a proper system with a well-defined internal rotation, which we will further call secondary relaxation mode. The structural design of the molecular rotors is ideal for this purpose, thus, we take advantage of a recently reported new class of glass-formers with a nonpolar sizable core and a small polar unit with high rotational freedom.^9,10^Figure 1 shows the chemical structure of the main compound used in our study. Structurally, three segments can be distinguished: (1) the rotating polar unit, (2) the core formed by diphenylamine and fluorene, and (3) alkyl chains that prevent crystallization and facilitate the formation of a stable glassy phase.

**Figure 1:**
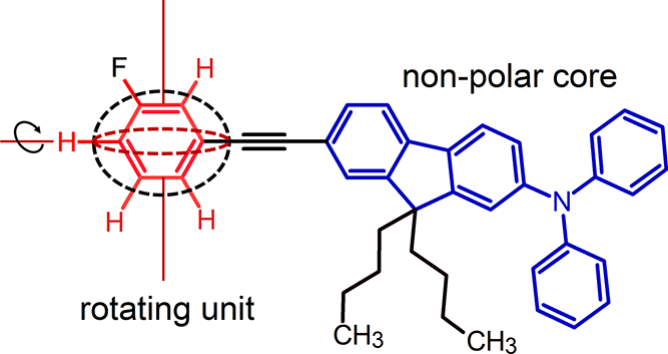
Chemical structure of M-meta-F.

We used a phenylene (Ph) ring as a mobile component (rotor) and as the source of the secondary relaxation discussed in our study. To ensure that the internal rotation of the Ph ring contributes to the dielectric response, we decorated the ring with a fluorine atom that introduced a dipole moment to the molecule. The dipole moment is a key quantity in dielectric research. Its change is imperative for dielectric detection of any relaxation process. By placing a fluorine atom at various positions of the phenylene ring, we were able to adjust the direction of the dipole moment in the molecules studied. The choice of fluorine to “rotor labeling” was not accidental because, due to its small size, the steric disturbances resulting from the replacement of hydrogen with fluorine have been reduced to a minimum. For this work, we selected a rotor with a fluorine atom attached to the Ph moiety at the meta position, *N*-(7-((3-fluorophenyl)ethynyl)-9,9-dibutyl-9*H*-fluoren-2-yl)-*N*,*N*-diphenylamine, referred to as M-meta-F (see Figure 1). This material and others discussed herein were synthesized by Trimen Chemical (Łódź, Poland).

In crystals, the mobility of the molecule as a whole is frozen, but in M-meta-F, the internal rotation of the phenylene unit remains active. This was possible due to the combination of several structural aspects that provide favorable molecular packing with enough free space around the Ph ring to realize the rotor’s motion in M-meta-F crystals. The concept is similar to those found in rotors embedded in rigid metal–organic frameworks (MOFs) which provide sufficient inner space for internal rotary motion.^11^ In our case, the free space was created by protruding pendant groups, mainly diphenylamine and alkyl chains, as indicated by single crystal X-ray diffraction data. The fragment of the crystalline array shown in Figure 2a illustrates how the Ph units of adjacent molecules are provided with free spaces between the static cores of sizable molecules, which allows internal rotation to be maintained in the crystalline state. Consequently, in M-meta-F crystals, we observed a relaxation process analogous to secondary relaxation in a glass.

**Figure 2:**
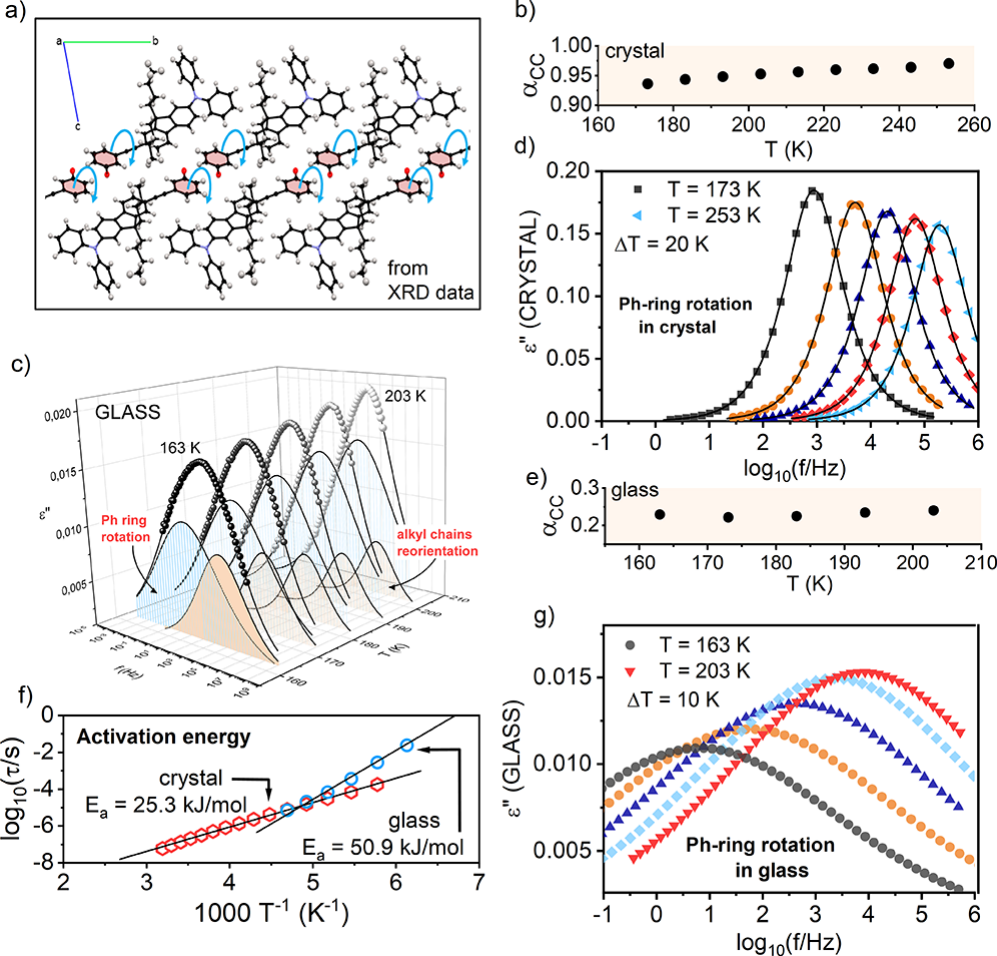
Dielectric investigation of Ph-unit rotation in M-meta-F at ambient pressure. (a) Molecular structure of the M-meta-F crystal. The α_CC_ parameters versus *T* for crystal (b) and glass (e). (c) Dielectric loss spectra for a glassy sample at various *T* with separated contributions to the dielectric response. The dielectric response originated from the internal rotation of the Ph unit in crystal (d) and glass (g). (f) Temperature dependence of relaxation times with corresponding values of activation energy.

To characterize the rotation of the Ph unit in the M-meta-F crystal with the BDS method, we recrystallized the M-meta-F sample directly in the BDS spectrometer between capacitor plates. Obtained polycrystalline samples were then subjected to dielectric measurements.^12^ The ε″(*f*) spectra revealed a single relaxation process with a narrow and symmetrical distribution of relaxation times, τ. The parametrization of ε″(*f*) data could be successfully carried out using the single Cole–Cole function (dashed lines in Figure 2d): ε*(ω) = ε_∞_ + Δε/[1 + (*iωτ*_CC_)^*α*_CC_^], where , Δε is dielectric strength, τ_CC_ is a Cole–Cole relaxation time related to the position of maximal loss, and α_CC_ is a shape parameter (α_CC_ = 1 corresponds to the Debye function).^13^ In the M-meta-F crystal, the rotation of the Ph ring occurs in a well-defined frame of reference created by the nearest neighbors and repeated translationally. Therefore, the observed relaxation mode revealed quasi-Debye behavior with *α*_CC_ ranging from 0.97 to 0.94 (Figure 2b). Referring to the previously mentioned comparison of the internal rotation of the Ph unit with a molecular probe, in this case, mapping refers to a repetitive and well-defined environment created by the periodic crystalline structure. Thus, the parameters obtained for the relaxation process in the crystalline state become a reference point for further discussion of measurements in which the environment of the rotating Ph unit will be modified in various ways.

The first approach aimed at modifying the local environment of the Ph rotor was the material’s vitrification leading to a glassy solid where M-meta-F molecules were frozen in disordered orientations lacking long-range order. The collected dielectric data depicted in Figure 2c revealed that the disorder around the rotating unit significantly affected the M-meta-F dynamics, leading to much broader distributions of relaxation times in comparison to crystals. As demonstrated in Supporting Information Figure S1, the widely distributed ε″(*f*) spectra could not be satisfactorily described by a single Havriliak–Negami function.^12^ To parametrize them, we applied two CC functions most commonly used for the description of secondary relaxations, both inter- and intramolecular (see Figure 2c and better resolution data in Figures S2 and S3 in the Supporting Material(12)).

To better understand the bimodal dielectric response of glassy M-meta-F, we synthesized a series of structurally related rotors with various substituted Ph units to study their dielectric response below *T*_g_ more systematically. Comparative analysis shown in Figure 3a–d revealed that in a glassy M-meta-F internal rotation of the Ph unit contributes to the dielectric response as a slower secondary mode. Faster relaxation alone was observed in the dielectric response of derivatives with sterically blocked or dielectrically inactive Ph-ring rotation. The faster process has not been studied in detail, but its universal presence and activation energy of *E*_a_ = 25.7 ± 0.4 kJ/mol similar to relaxations observed in some alkylated glasses,^14^ suggested a possible relationship with chain dynamics.

**Figure 3:**
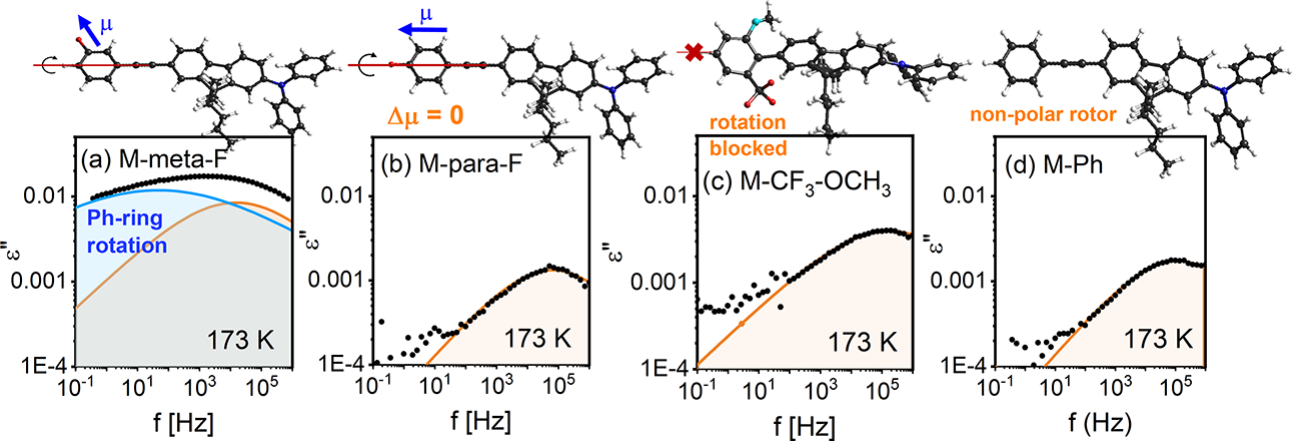
Comparison of the dielectric response of structurally related rotors with variously functionalized rotating units which differentiate molecules in terms of direction of the dipole moment vector μ, the range of rotational freedom, and the polarity of the rotor: (a) M-meta-F with μ = 2.5 D, (b) M-para-F with μ = 2.2 D directed along the long molecular axis, (c) M-PhCF_3_OCH_3_ where bulky substituents hinder internal rotation of Ph-ring, and (d) M-Ph with a nonfluorinated rotor. Only in M-meta-F is the internal rotation of the Ph ring dielectrically active.

When we successfully unraveled the bimodal dielectric response of glassy M-meta-F, in the next step, we could extract the part of the dielectric response related to the internal rotation of the Ph ring (by subtracting the faster relaxation’s contribution; see Figure 2g). For glassy samples, the broad distribution of relaxation times was reflected in lower values of the α_CC_ parameter (in the range of 0.22–0.24) in comparison to crystals. In addition, the value of α_CC_ was somewhat temperature-dependent, which can be inspected in Figure 2b (for crystal) and Figure 2e (for glass). In both cases, α_CC_ slightly decreases on cooling.

To discuss the secondary relaxation dynamics in terms of the distribution of energy barriers, we used the approach previously described by Nagel et al.,^15,16^ It allowed us to translate the collected ε″(f) data into Gaussian distribution of energy barriers centered around the *E*_a_ value with . Assuming that the wide dielectric response is due to many Debye relaxations, we get the following log-normal function:^17^
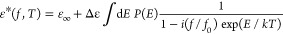
The energy width parameter σ is related to the frequency width parameter *W* as σ/*k* ≈ *WT* ln(10), where *k* is a Boltzmann constant and *W* is the 1/e half-width of the ε″(*f*) peak assessed in Figure S4 in the Supporting Material.^12^ For crystals, we found quasi-Debye behavior, but the broad and non-Debye dielectric response of glass could be analyzed in terms of the Gaussian distribution of barrier heights. The concept we want to show is based on mapping of local interactions through the internal rotation of Ph units. Consequently, parameters describing the rotor mobility mirror the molecular-level properties of the environment. When Ph units rotate in ideal crystalline surroundings, they are all characterized approximately by the same single barrier. When motion takes place in a glass, the disorder in the rotor’s vicinity leads to a large distribution of barrier heights. In both cases, the barriers are due to specific “contacts” between H and F as revealed based on DFT analysis. It is worth mentioning that, in systems with an ordered crystal lattice, a departure from the Debye behavior manifested as a broadening of the dielectric loss peak can be also observed (e.g., for plastic crystals,^18^ or some MOFs^19−21^). For dipolar molecules, this can be attributed to dipole–dipole interactions. In the M-meta-F crystal, the dipoles seem to be sufficiently separated to limit interdipolar interactions; thus, this effect was less significant. Figure 4a shows the σ values calculated for the internal rotation in a glassy state. In the *T* range covered by our study, the width σ was found to be weakly temperature-dependent. The remarkably different and environment-sensitive behavior of intramolecular relaxation is nicely illustrated in Figure 4b, where the distribution of relaxation times and the corresponding distribution of energy barriers (Figure 4c) were calculated from Cole–Cole parameters.^22^ The differences observed for ordered and disordered surroundings showed that rotation of the Ph unit is very sensitive to the environment and thus cannot be treated as a trivial internal motion of little importance due to its intramolecular origin and the fact that only part of the molecule is involved.

**Figure 4:**
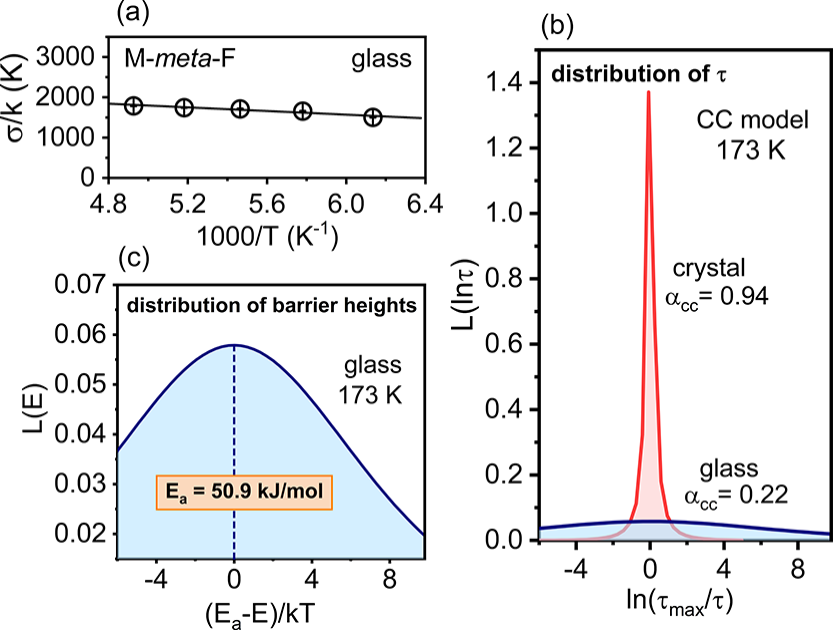
Distribution of energy barriers detected by internal rotation of Ph unit in a glassy state. (a) σ parameter as a function of *T*^–1^. (b) Distribution of relaxation times calculated at 173 K from the parameters of the CC model: .^22^ In a crystal, the distribution is heavily peaked around the τ_max_ value. In contrast, the very broad distribution of τ around τ_max_ was observed in glass. (c) Corresponding broad distribution of energy barriers around the *E*_a_ value in a glass.

To determine the activation parameters for internal rotation in M-meta-F, we used the relaxation times τ_max_ corresponding to the maximum of the ε″(*f*) peak determined from the Cole–Cole fitting parameters. The relaxation times follow Arrhenius temperature dependence with activation energy equal to *E*_a_ = 25.3 ± 0.2 kJ/mol and prefactor log τ_0_ = −11.4 ± 0.1 s for crystal, and *E*_a_ = 50.9 ± 1.6 kJ/mol and log τ_0_ = −17.8 ± 0.5 s for glass (Figure 2f). These results demonstrated that changing the local environment impacts not only the distribution of energy barriers but also the magnitude of the barrier height. The fact that, in a less dense glassy state, the *E*_a_ value increased 2-fold points out the role of disorder. In a crystalline environment, the rotor can move more independently in the free spaces created by the orderly arranged neighbors, as shown in Figure 2a. In a disordered glassy environment, the random arrangement of molecules inhibits rotation, leading to higher rotational barriers. The observed differences indicated also that in the studied system the activation energy depends not only on intra- (internal rotational barriers) but also intermolecular (the environment of the relaxing unit) contributions.^23^ According to performed DFT calculations^24^ utilizing the hybrid B3LYP functional,^25^^26,27^ and the def2-SVP basis set,^28^ the barrier associated with the Ph-unit rotation in an isolated M-meta-F molecule is 4.7 kJ/mol. The much higher value observed experimentally is due to the importance of the intermolecular contributions. This behavior can be understood by considering that a fluorine atom attached to the rotating unit can interact with neighboring molecules through weak C–H···F contacts. Each contact of fluorine and hydrogen affects the rotation parameters and is a source of information about the rotor surroundings. To some extent, this behavior is similar to that of an atomic force microscope (AFM) probe, but in this case, scanning the surroundings is accomplished through intermolecular contacts between fluorine and hydrogen atoms during rotation. Such behavior can underlie the unprecedented sensitivity of internal dynamics to the environment in investigated material where the rotating units can probe the local interactions with nm-scale precision.

The second approach used to change the local environment around the rotating unit was the application of isothermal compression. It is worth mentioning that the pressure sensitivity of secondary dynamics is often used by researchers as justification for the intermolecular origin of a secondary process.^29^ Accordingly, processes with pressure-dependent peak frequency are usually classified as intermolecular Johari–Goldstein (JG) relaxations with fundamental importance and connection to structural relaxation and glass transition.^4,30^ Their universal presence in different types of glasses has been discussed for years.,^31−33,30,34,35^ In the M-meta-F the situation is clear. The discussed process is due to the internal rotation of the Ph unit and involves intramolecular degrees of freedom. Since the analogous relaxation was observed in the crystal, it cannot be a JG relaxation.

The relaxation data collected during the isothermal compression of glassy and crystalline M-meta-F at 263 K are presented in Figure 5c,d.^12^ In both cases, the maximum of the loss peak ε″(*f*) shifts during compression. Although the mobility of the Ph unit involves intramolecular degrees of freedom, modification of the local environment through compression clearly affects its internal dynamics. Figure 5e shows the pressure dependence of relaxation times τ_max_ parametrized with volume activation law, τ_max_ = τ_0_ exp(*P*Δ*V*^#^/*RT*), where Δ*V*^#^ is activation volume and *R* is a gas constant. The value of Δ*V*^#^ found for the M-meta-F crystal (Δ*V*^#^ = 37.1 ± 0.2 cm^3^/mol) and glass (Δ*V*^#^ = 36.3 ± 0.6 cm^3^/mol) was comparable and relatively large, demonstrating a significant sensitivity of the rotational dynamics to compression. Again, such behavior is not typical for internal modes, which usually are characterized by no or little sensitivity to compression.,^3,36^ For comparison, several examples can be invoked here: i.e., PPGA and DGEBA (JG type) with Δ*V*^#^ = 15.0 cm^3^/mol (293 K)^37^ and Δ*V*^#^ = 21.2 cm^3^/mol (293 K);^38^ PDE and BMPC (non-JG type) with Δ*V*^#^ = 21.3 cm^3^/mol (293 K)^38^ and Δ*V*^#^ = 5.1 cm^3^/mol (260 K),^39^ respectively.

**Figure 5:**
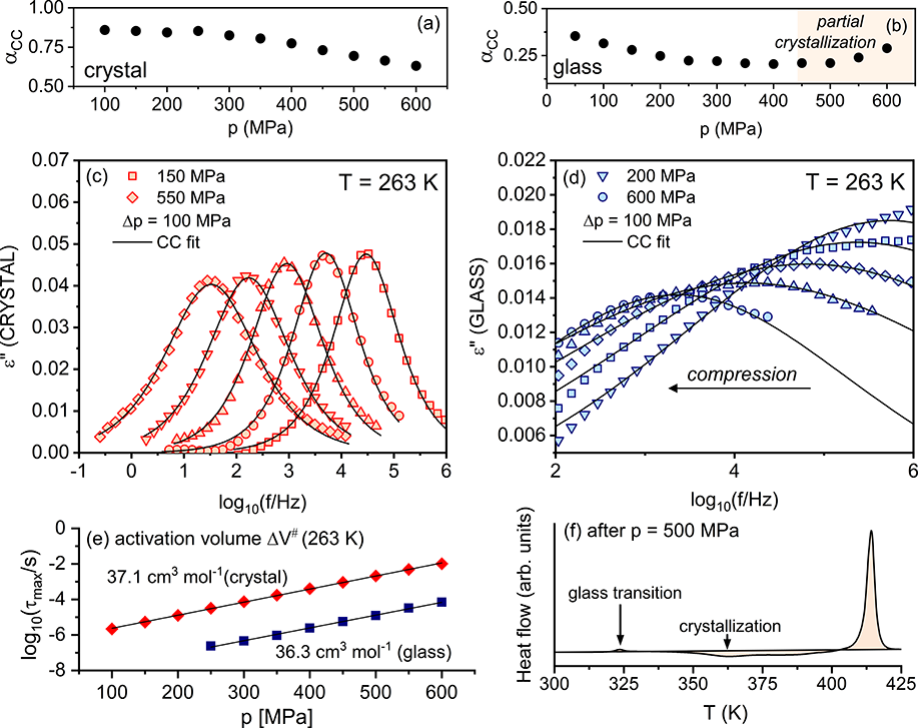
Dielectric investigation of internal rotation in M-meta-F at elevated pressure. Pressure dependence of the α_CC_ parameter for crystal (a) and glass (b). Representative loss spectra at 263 K measured during compression of crystalline (c) and glassy (d) M-meta-F. (e) Pressure dependence of τ_max_ with indicated Δ*V*^#^ values. (d) Differential scanning calorimetry (DSC) scan measured during heating (10 K/min) of glassy M-meta-F after compression to 500 MPa showing partial crystallization of the sample (endo up).

The analysis of ε″(log *f*) data with the CC function showed that, up to 600 MPa, the value of the α_CC_ parameter was in the range of 0.86–0.63 (at high *p*) for crystal and 0.36–0.20 for glass. In the crystal, the width parameter systematically dropped during compression, while, in the glass above 450 MPa, it began to increase (Figure 5a,b). The calorimetric measurements of squeezed glasses showed that the narrowing of the ε″(*f*) peak was due to the partial crystallization of the sample (see Figure 5f) initialized by a value of pressure of order 450 MPa. It means that the partial ordering of the rotor surrounding was successfully detected by rotating the Ph unit. It proves again the exceptional sensitivity of internal dynamics to intermolecular contributions.

Our results show that in investigated rotor molecules the internal rotation, like a probe, can sample the immediate surroundings with high sensitivity to molecular-level changes that impact the rotation parameters. The importance of secondary relaxations that do not involve whole molecules but only molecular subunits has often been marginalized. However, in our system, the conservation of the internal rotation was neither trivial nor insignificant. Secondary dynamics exhibited by rotor molecules differ from the picture attributed to the internal mobility of the molecular side groups. This striking dynamic behavior can contribute to the ongoing discussion of the phenomenology of secondary processes and the universality of their behavior. The unconventional vision of sampling the environment through internal rotation is intriguing for further exploration by the dielectric community.

## Supporting Information Available

Experimental details of dielectric measurements; supporting analysis of ε″(*f*) data for the glassy sample (PDF)
