# Supplementary material for: Internal Secondary Relaxation as a Dielectric Probe of Molecular Surroundings
Source: J Phys Chem Lett. 2024 Feb 28;15(9):2595–600. doi: 10.1021/acs.jpclett.4c00128 (PMC10926159; doi:10.1021/acs.jpclett.4c00128)
Supplement: Supplementary file 1 — jz4c00128_si_001.pdf [file jz4c00128_si_001.pdf]

Supporting Information

Internal Secondary Relaxation as a Dielectric Probe of Molecular Surroundings

Marzena Rams-Baron*, Alfred Błażytko, Maria Książek, Joachim Kusz, Marian Paluch
August Chelkowski Institute of Physics, University of Silesia in Katowice,
75 Pulku Piechoty 1, 41- 500 Chorzow, Poland
*corresponding author: marzena.rams-baron@us.edu.pl

1. Experimental details of dielectric measurements

Dielectric measurements at ambient pressure were performed using a Novo-Control GMBH Alpha dielectric spectrometer (Germany). When the polycrystalline sample was measured, the temperature (173K – 293 K) was controlled with the Novocool Cryosystem (with accuracy ± 0.3 K). For the glassy sample, the temperature stabilization within the range 163 K – 203 K was provided by Quatro Cryosystem (stabilization accuracy ± 0.1 K). Measurements were performed in the frequency range from 10^{-1} Hz to $3 \cdot 10^{6}$ Hz. We used a stainless-steel parallel-plate capacitor (15 mm in diameter; with a 0.1 mm gap provided by fused silica fibers). Glassy samples were vitrified between capacitor plates before the BDS measurement (melting on a hot plate and fast transfer below T_g using a chilled copper plate). The melting temperatures determined from differential calorimetric measurements were T_m = 415 K for M-meta-F, T_m = 405 K for M-Ph, T_m = 414 K for M-para-F, and T_m = 419 K for M-$PhCF_3OCF_3$. To prepare crystalline samples, the material was recrystallized *in situ* between capacitor plates at 373 K.
For high-pressure measurements up to 600 MPa, the samples were placed in a high-pressure chamber and compressed using silicon oil. We used a high-pressure setup designed by Unipress (Warszawa, Poland) connected to impedance analyzer Alpha-A by Novocontrol (Germany). The samples were placed in a parallel-plate capacitor (15 mm diameter and 0.1 mm gap with a Teflon spacer) with cables providing electrical contact with the analyzer. High pressure was generated by a U111 pump. The pressure was measured using a Nova Swiss tensometric meter with a resolution of 0.1 MPa. To avoid contamination with the pressure-transmitting fluid, the capacitor with a sample was secured with Teflon tape. The temperature of 263 K was provided by the Julabo heating circulator (Seelbach, Germany).

2. Supporting graphics for the fitting analysis of ε"(f) data for the glassy sample

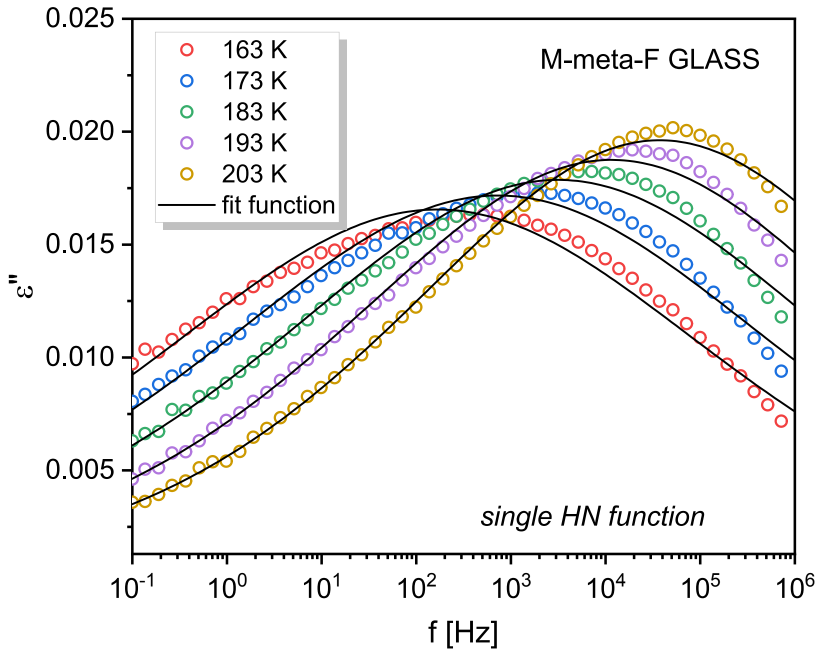

Figure S1. Fitting analysis based on single Havriliak-Negami functions.

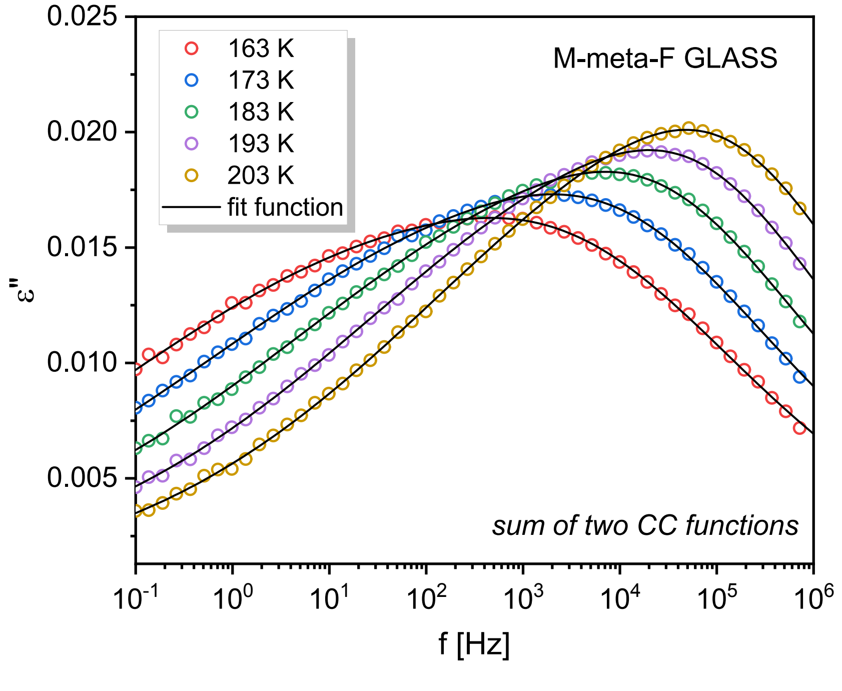

Figure S2. 2D representation of data from Fig. 2 showing fitting analysis based on two Cole-Cole functions.

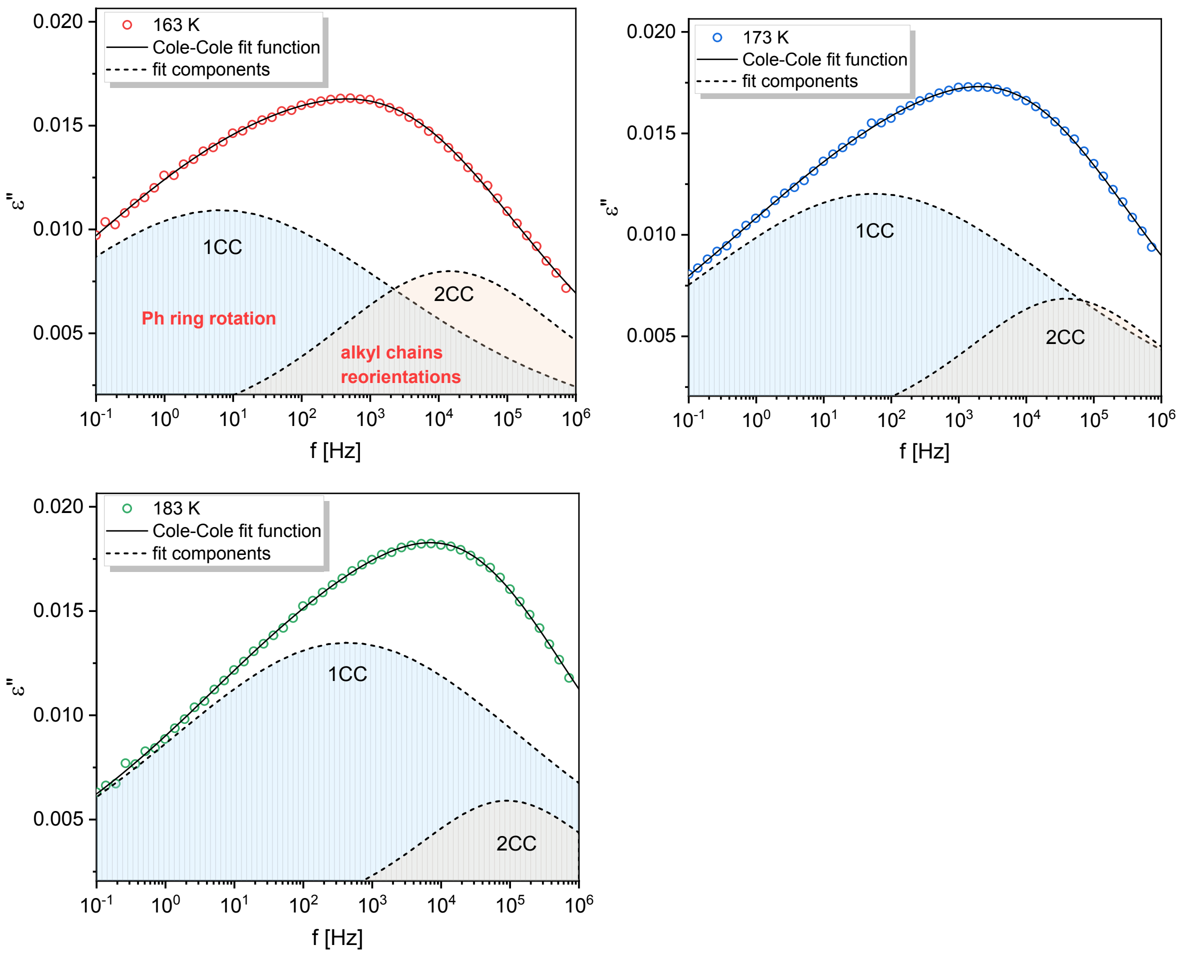

Figure S3. 2D representation of representative data from Fig. 2 showing fitting analysis based on two Cole-Cole functions with fitting components.

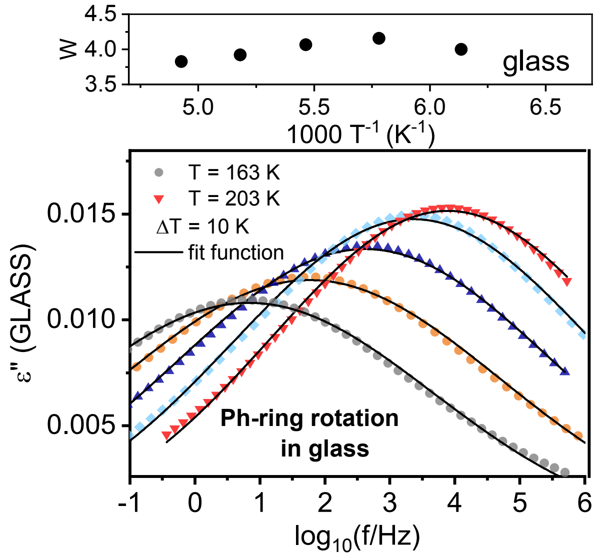

Figure S4. The ε"(f) data (bottom panel) for M-meta-F in the glassy state fitted with log-normal function $\varepsilon''(f) = \frac{\Delta\varepsilon}{\sqrt{\pi}W}\exp\left[-\left(\log_{10}f - \log_{10}f_{max}\right)^2/W^2\right]$ where f_{max} corresponds to the maximum of the ε"(f) peak and W is the 1/e half-width of the peak. The upper panel shows the temperature evolution of the W parameter.
